# Material discrimination and mixture ratio estimation in nanocomposites via harmonic atomic force microscopy

**DOI:** 10.3762/bjnano.8.276

**Published:** 2017-12-21

**Authors:** Weijie Zhang, Yuhang Chen, Xicheng Xia, Jiaru Chu

**Affiliations:** 1 Department of Precision Machinery and Precision Instrumentation, University of Science and Technology of China, Hefei 230026, China

**Keywords:** atomic force microscopy, harmonic imaging, material discrimination, mixture ratio, nanocomposites

## Abstract

Harmonic atomic force microscopy (AFM) was employed to discriminate between different materials and to estimate the mixture ratio of the constituent components in nanocomposites. The major influencing factors, namely amplitude feedback set-point, drive frequency and laser spot position along the cantilever beam, were systematically investigated. Employing different set-points induces alternation of tip–sample interaction forces and thus different harmonic responses. The numerical simulations of the cantilever dynamics were well-correlated with the experimental observations. Owing to the deviation of the drive frequency from the fundamental resonance, harmonic amplitude contrast reversal may occur. It was also found that the laser spot position affects the harmonic signal strengths as expected. Based on these investigations, harmonic AFM was employed to identify material components and estimate the mixture ratio in multicomponent materials. The composite samples are composed of different kinds of nanoparticles with almost the same shape and size. Higher harmonic imaging offers better information on the distribution and mixture of different nanoparticles as compared to other techniques, including topography and conventional tapping phase. Therefore, harmonic AFM has potential applications in various fields of nanoscience and nanotechnology.

## Introduction

Because of its extreme importance and widespread applications, nanocomposites have received more and more attention [1]. Nanocomposites are usually composed of a bulk matrix and some nanofillers. The fillers can be nanoparticles (NPs), nanotubes, nanorods, nanosheets and so on. The physical and chemical properties of nanocomposites will often differ from their component materials and show remarkable added functionalities [2]. The characterization of the distribution and mixture ratio of two or more different components at nanoscale resolution is of particular significance because the materials are closely related to the functional performance [3].

Scanning/transmission electron microscopy is commonly applied for the detection of mixed NPs [4]. However, it is rather difficult to distinguish a mixture of NPs having similar geometry and dimensions from topographical electron microscopy images. On the contrary, atomic force microscopy (AFM) methods can simultaneously characterize physical and chemical properties in addition to the topographic information [5–10]. The materials can be consequently identified by differences in the measured physical characteristics, and in particular, discrimination through mechanical properties has broad potential applications. These operation modes including tapping phase, force modulation mode and contact resonance AFM have their own features and ideal conditions [11–13]. For example, the phase signal in tapping AFM carries certain information on the mechanical properties. Such a mode reduces sample damage and allows softer materials to be nondestructively scanned. However, the interpretation of tapping phase results is rather complex because many factors may influence the results [14–15]. For force modulation and contact resonance operations, the tip is maintained in contact with the sample during the scan while the cantilever oscillations are monitored. The amplitude in force modulation and resonance frequency in contact resonance AFM are used to extract the mechanical properties quantitatively [16–17]. However, the continuous tip–sample contact may cause severe sample damage or tip wear.

In tapping mode, the tip can touch the sample periodically. Due to the nonlinear contact force, the cantilever oscillation will show certain higher harmonic components [18], which are dominated by contact force and contact time [19–21]. Theoretical analysis and preliminary experiments have demonstrated that the harmonic signals are highly sensitive to the variation of local elasticity [22]. In addition, unlike force modulation and contact resonance techniques, the tip–sample contact time is dramatically reduced, which is suitable for imaging soft samples. In this regard, one of the most appealing characteristics of harmonic AFM is that it provides a method for fast, high-resolution and nondestructive mechanical mapping of numerous specimens [23–26].

Here, we employed harmonic AFM to distinguish different materials, visualize the distribution of NPs in composites, and estimate the mixture ratio in a quantitative manner. First, some important factors affecting harmonic signals were investigated systematically on a polymer blend composed of polystyrene (PS) and low-density polyehtylene (LDPE). We focused on the influence of feedback amplitude set-point, drive frequency and laser spot position along the cantilever beam. Based on these fundamental studies, harmonic AFM imaging was utilized to distinguish NP mixtures with different elastic properties and mixture ratios.

## Results and Discussion

### Effect of amplitude set-point

**Figure 1:**
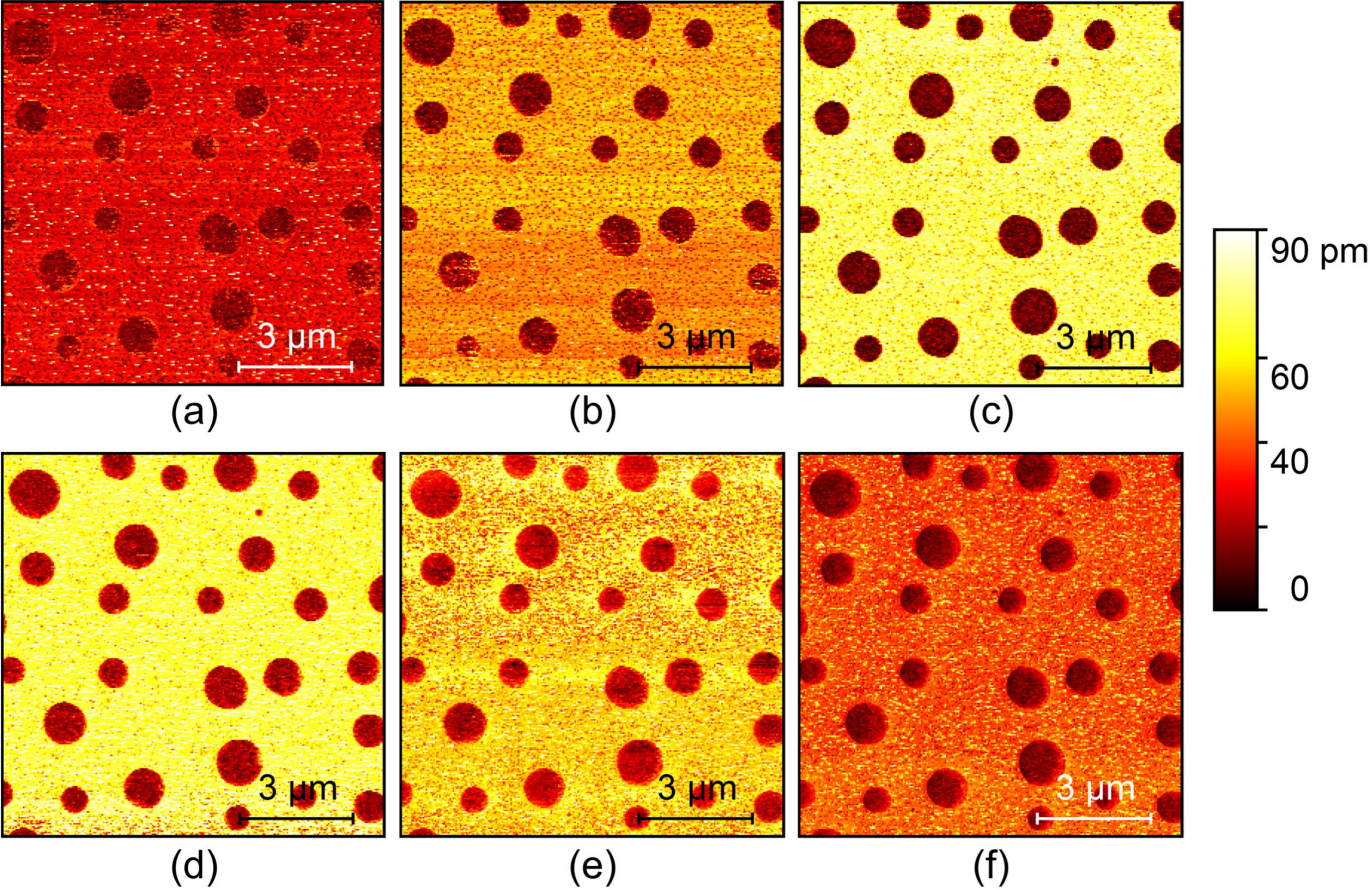
Typical sixth harmonic amplitude images of a PS/LDPE blend with different amplitude set-points. (a)–(f) The set-points are 0.4, 0.35, 0.3, 0.25, 0.2 and 0.15, respectively.

The first investigated factor is the amplitude set-point, which is specified by *A*/*A*_0_ where *A* is the feedback amplitude and *A*_0_ the free amplitude. In tapping mode AFM, the contact force and contact time per oscillation period dominate the harmonic signals [27]. These two quantities are assumed to be related to the amplitude set-point. It is well known that the peak force in tapping mode scales with the amplitude feedback settings [28]. However, the effect of amplitude set-point in harmonic AFM imaging remains unclear. In experiments, the sample was a PS/LDPE blend with a nominal elastic moduli of *E*_PS_ ≈ 2 GPa and *E*_LDPE_ ≈ 100 MPa. The calibrated cantilever free amplitude and spring constant were 167 nm and 0.32 N/m, respectively. The first two free resonance (FR) frequencies were measured to be 15.13 kHz and 95.15 kHz. Then, the amplitude set-point was decreased from 0.4 to 0.15 with a step of 0.05 while other settings were kept the same. The amplitude image at the sixth harmonic of the fundamental resonance frequency was mainly concerned because it showed stronger signal strengths than other harmonics for a rectangular cantilever [29]. When the set-point was larger than 0.4, the harmonic images were not suitably clear and stable in our experiments. Figure 1 shows typical amplitude images at the set-points of 0.4, 0.35, 0.3, 0.25, 0.2 and 0.15. Two conclusions can be drawn from direct observation. First, higher harmonic amplitudes appear on the PS domain (brighter area), which has a higher elastic modulus. Second, the amplitude difference between PS and LDPE first increases and then decreases when decreasing the set-point within the experimental range.

**Figure 2:**
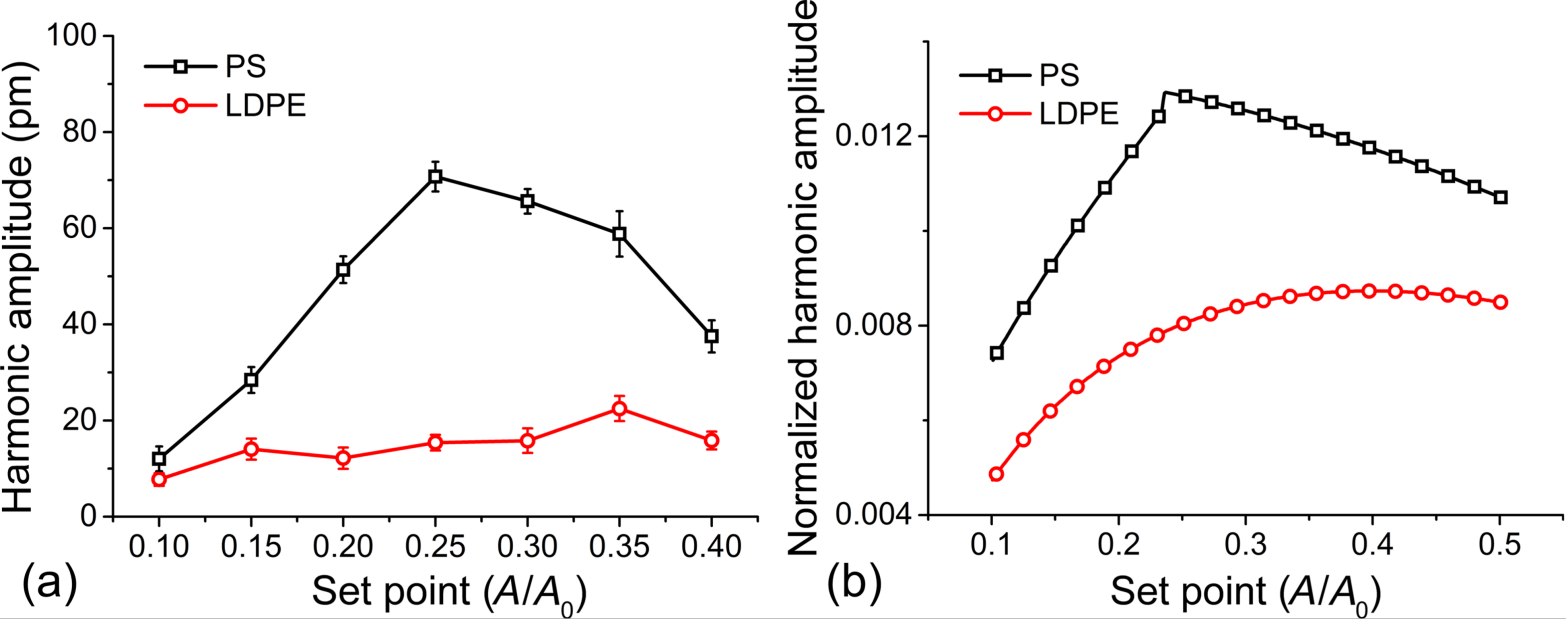
Dependence of the sixth harmonic amplitude on the set-point. (a) Experimental results. (b) Numerical results.

For quantitative analysis, we evaluated the harmonic amplitudes on the PS and LDPE domains, and the results are shown in Figure 2a. The amplitude differences were 4.3, 14.4, 39.2, 55.4, 49.8, 36.5 and 21.7 pm when the set-point was increased from 0.1 to 0.4 with an interval of 0.05. In addition to the amplitude difference, image contrast is another important issue among different materials. In the evaluation, the following contrast metric was employed [30],

[1]
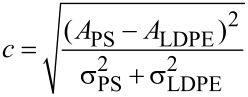


where *A* and σ^2^ are the mean and variance of the amplitude and the subscripts correspond to the PS and LDPE domains. The higher this metric value is, the better the material discrimination. The corresponding contrast values are 0.9, 3.4, 9.8, 11.5, 10.2, 6.5 and 5.5. It is obvious that the harmonic image has the largest contrast and amplitude difference at the set-point of approximately 0.25.

To interpret and ascertain the experimental dependence of the sixth harmonic amplitude on the set-point, a theoretical simulation of the harmonic imaging was carried out. The cantilever dynamics in the presence of the tip–sample interaction forces can be described by [31],

[2]
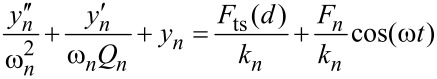


In Equation 2, the integer number *n* refers to the *n*th eigenmode and *y_n_* denotes the cantilever deflection. *F_n_* = *k_n_A_n_*/*Q_n_* is the drive force. *k_n_*, *A_n_*, *Q_n_* and ω*_n_* are the equivalent cantilever stiffness, amplitude, quality factor and angular resonance frequency, respectively. The interaction forces *F*_ts_, depend on the instantaneous gap, *d*, and they are simplified as,

[3]
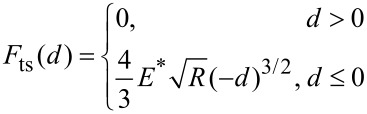


Here the attractive forces are neglected. *R* is the tip radius and E^*^ is the reduced modulus


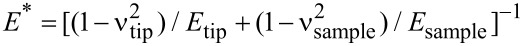


where *v* Poisson’s ratio. Combining Equation 2 and Equation 3 and utilizing numerical methods such as Runge–Kutta algorithms, the real-time oscillation trajectory of the tip can be achieved. The above contact model is based on the main assumption that the tip–sample contact is purely elastic. Though such a mode greatly simplifies the practical situations, it can provide reasonable interpretation of the experimental results. The harmonic amplitude at a specified frequency is further obtained by using the Fourier transformation. We analyzed the sixth harmonic amplitude at different set-points as presented in Figure 2b. The main parameters used for the calculation are listed in Table 1, which are selected according to typical experimental conditions. For simplicity, only the first two eigenmodes are considered in Equation 2. Simulations verify that the harmonic response depends on the contact time and peak contact force. Both quantities are related to the elasticity of the sample. Because the elastic modulus of PS is larger than that of LDPE, oscillating on the PS domain produces a much shorter contact time and a larger peak force in each oscillation cycle. As a result, the harmonic amplitude on PS is larger. The general trend of the amplitude as a function of set-point agrees well with the experimental one. With the increase of the set-point from 0.1 to 0.24, both the amplitude magnitude and amplitude difference increase. Over a threshold of approximately 0.24, the amplitude difference starts to decrease. Such a characteristic has also been noted in a previous publication. The higher harmonics were found to have an inverted U-shape dependence on the tip–sample gap [32]. Despite the deviation in the quantitative values, the experimental and numerical curves are in reasonable accordance with each other. Simulating the cantilever dynamics can enable proper selection of the set-point for better harmonic imaging. In addition, it helps to interpret the imaging process because the underlying parameters such as real-time interaction forces and contact time are not directly accessible in experiments. On the contrary, they can be exported for further analysis in numerical simulations if necessary.

**Table 1:** Main parameters used in the harmonic atomic force microscopy simulations.

drive frequency	15.13 kHz
cantilever free amplitude	167 nm
cantilever spring constant	0.32 N/m
first eigenfrequency	15.13 kHz
second eigenfrequency	95.15 kHz
first eigenmode quality factor	80
second eigenmode quality factor	160
tip radius	20 nm

### Effect of drive frequency

**Figure 3:**
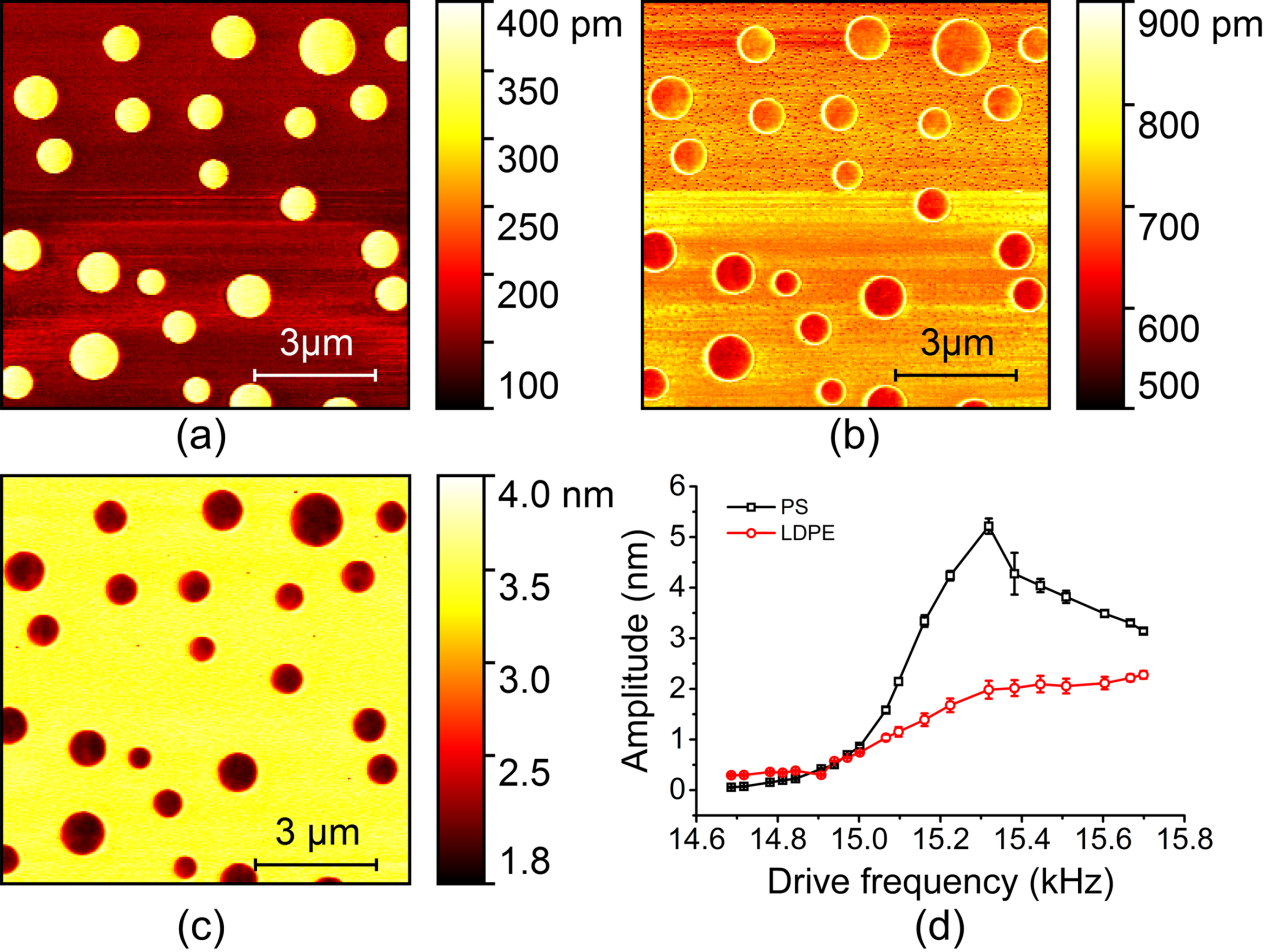
Influence of drive frequency on the sixth harmonic amplitude. The fundamental free resonance frequency of the cantilever is 15.13 kHz. (a)–(c) Harmonic amplitude images at the drive frequencies of 14.78 kHz, 14.97 kHz and 15.60 kHz, respectively. (d) Harmonic amplitudes on the PS and LDPE domains when the drive frequency is altered.

The higher harmonic amplitudes in our experiments are analyzed with a lock-in amplifier at the integer multiples of the drive frequency, which is usually selected at the fundamental resonance. The discrepancy between harmonic and higher-mode resonance frequencies affects the harmonic amplitude. Thus, the accurate selection of the drive frequency is of vital importance. We performed a set of harmonic imaging experiments at slightly different excitation frequencies. The cantilever was the same as used previously. In experiments, the drive frequency was varied from 14.68 kHz to 15.70 kHz passing through the first FR frequency of 15.13 kHz while other settings were kept the same. The sixth harmonic amplitude images were recorded as shown in Figure 3. When the drive frequency is slightly lower than the FR frequency, the harmonic amplitudes on PS and LDPE are 155 ± 9 pm and 358 ± 12 pm, respectively, see Figure 3a. The amplitude on the PS domain with a higher modulus is even lower than the LPDE domain, indicating the appearance of contrast reversal. The behavior is in contrast with the results presented in Figure 1. If the drive frequency is changed to about 14.97 kHz, the harmonic amplitudes on PS and LDPE are 690 ± 9 pm and 680 ± 8 pm, respectively, see Figure 3b. The signal magnitudes increase slightly but it is rather difficult to distinguish the two materials owing to the small amplitude difference. When the drive frequency is larger than the FR frequency, the harmonic amplitudes on PS and LDPE are 3.49 ± 0.05 nm and 2.11 ± 0.09 nm, respectively, see Figure 3c. The amplitude magnitude and the amplitude difference are nearly optimal. The relations between the harmonic amplitude and the drive frequency of the two materials are shown in Figure 3d. Improved harmonic signal contrast can be achieved when the drive frequency is at or slightly larger than the first FR frequency. Furthermore, amplitude image reversal emerges when the drive frequency increases from below to above the FR frequency.

**Figure 4:**
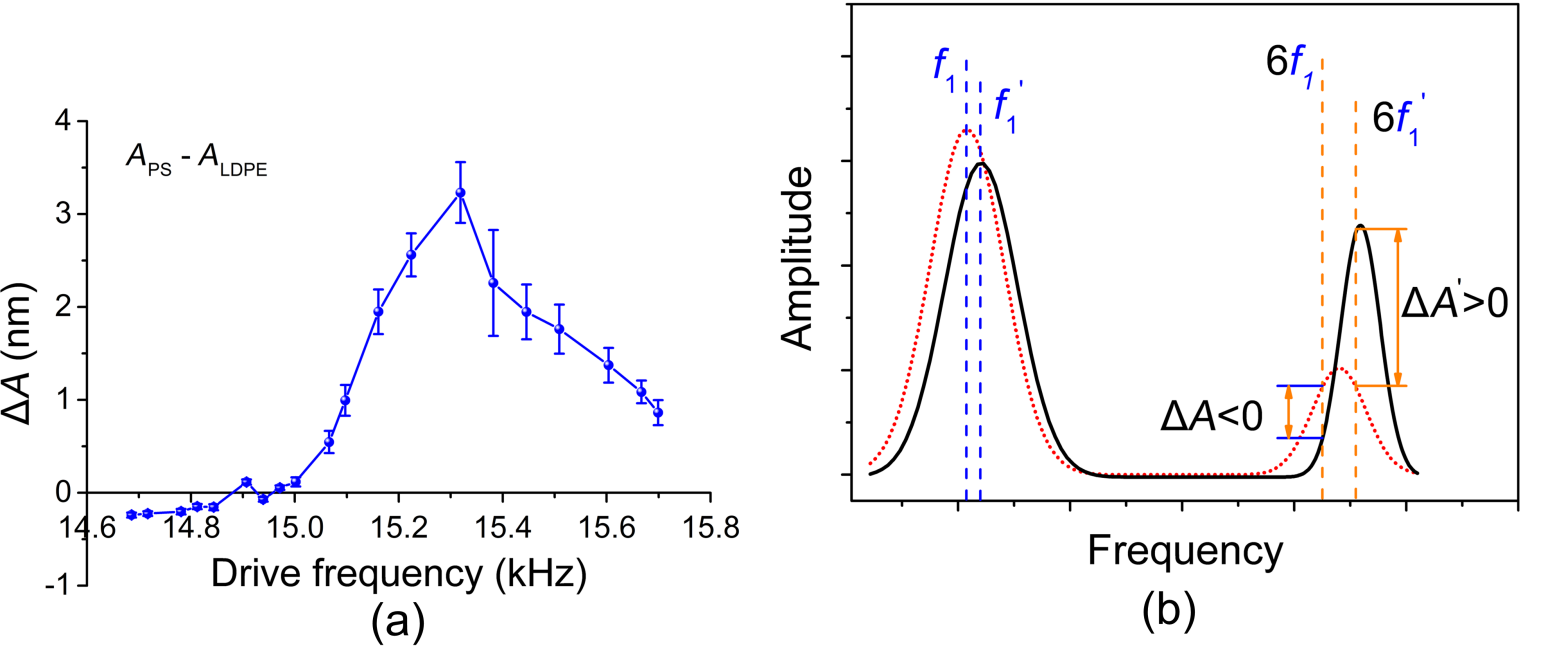
Harmonic amplitude contrast reversal when the drive frequency is altered. (a) Amplitude difference between PS and LDPE domains. (b) Schematic interpretation of the contrast reversal. The black solid curve illustrates the frequency spectrum on the stiffer material, PS, and the red dotted curve denotes the spectrum on the softer material, LDPE. The first two eigenfrequency peaks are schematically shown here.

The dependence of the experimental amplitude difference (Δ*A* = *A*_PS_ – *A*_LDPE_) on the cantilever drive frequency is evaluated in Figure 4a. At a relatively lower drive frequency, the difference yields a negative value. However, at a higher drive frequency, the amplitude difference becomes positive. The results clearly demonstrate the presence of contrast reversal, which may cause difficulty in further establishment of the quantitative relation between harmonic amplitude and local elastic modulus. To realize this objective, precise determination of the first FR frequency and drive frequency may be required. Such a reversal phenomenon is reasonable. At the contact time duration in each tapping period, the repulsive interactions between the tip and sample can shift the cantilever resonances to higher frequencies [33–34]. A higher elastic modulus, and therefore higher stiffness, will lead to a larger frequency shift, as schematically illustrated by the solid line, see Figure 4b. Similarly, a smaller elastic modulus causes a smaller frequency shift, as depicted by the dotted line. When the drive frequency is set at a relatively lower frequency *f*_1_, the amplitude at the harmonic frequency 6*f*_1_ on the softer LDPE will be larger than that on the stiffer PS. In contrast, when the drive frequency *f*_1_^′^ and the corresponding harmonic 6*f*_1_^′^ are larger, the amplitude difference will be positive, that is, harmonic amplitude contrast reversal occurs. The contrast reversal with an increase of the drive frequency can thus be qualitatively interpreted. From the cantilever spectra we find that the resonance frequency shift and the quality factor induce the contrast inversion. Actually, the contrast reversal in the harmonic amplitude image is quite similar to that observed in contact resonance AFM. Further details can be found in [35]. In the quantitative mapping of the harmonic response to the local elastic properties this factor has to be taken into account. Owing to the complexity in each influencing factor, the utilization of reference materials may reduce the difficulty in mathematical modeling. However, this subject requires further numerical and experimental studies and is beyond the scope of current work.

### Effect of laser spot position

**Figure 5:**
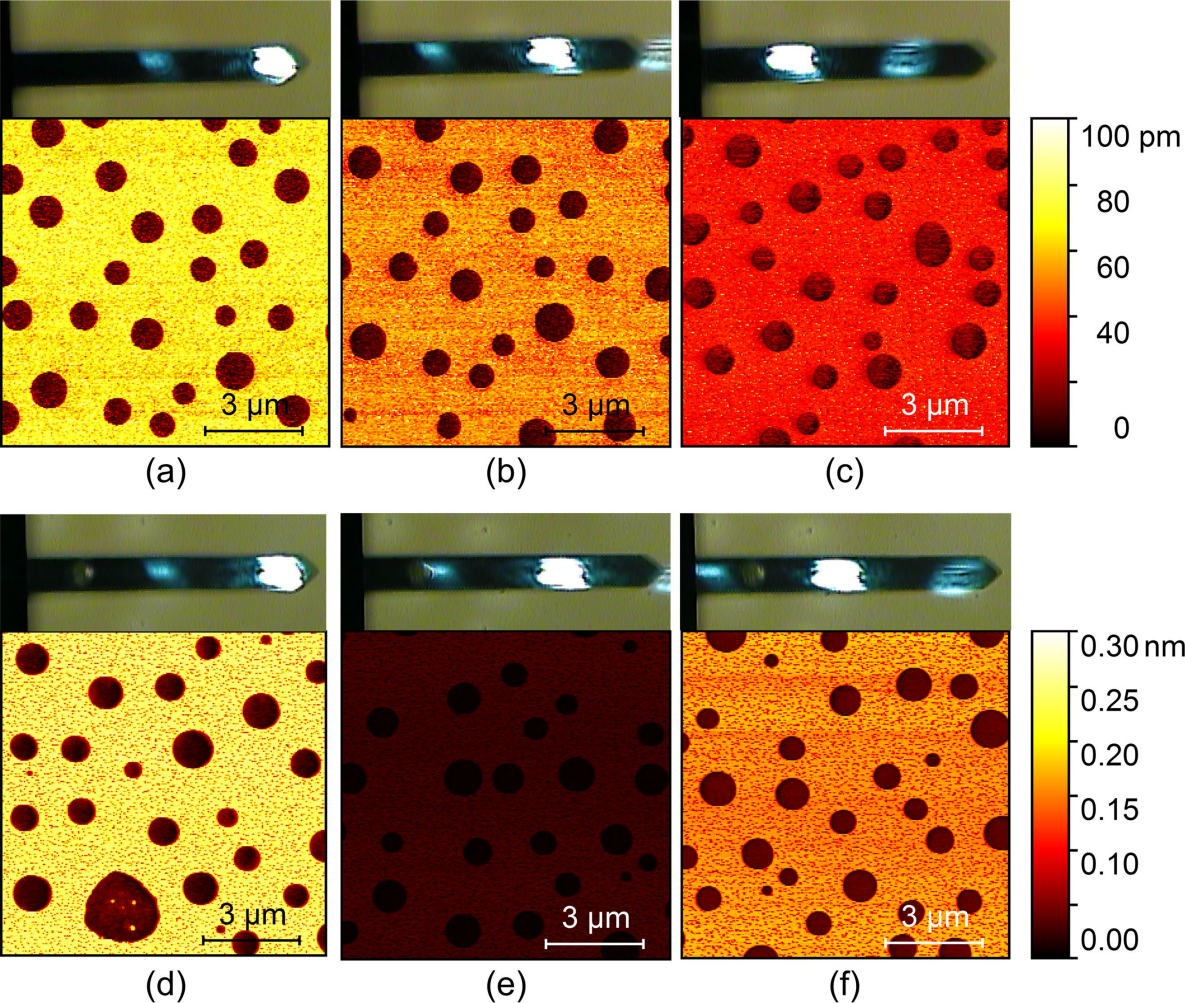
Typical harmonic amplitude images when the laser spot location on the cantilever beam is changed. The laser spot positions are estimated from the optical microscopy images. (a)–(c) The 6th harmonic amplitudes. (d)–(f) The 17th harmonic amplitudes. The 6th and 17th harmonic frequencies are respectively near the second and third eigenmode resonance frequencies.

In most AFM instruments, the cantilever deflection is measured by the optical beam deflection method. It is well known that optical lever sensitivity depends on the laser spot position along the cantilever beam axis [36]. To ascertain the influence of the laser spot position on the harmonic signals, we moved the laser spot from the free end to the fixed end gradually and measured the 6th and 17th harmonic amplitudes. These two harmonic frequencies are respectively close to the second and third eigenmode resonance frequencies. The laser spot location is estimated from the optical monitor integrated with the AFM system. Some typical results are depicted in Figure 5. With the change of the laser spot position, the harmonic images have significant differences, as expected. The 6th (Figure 5a–c) and the 17th (Figure 5d–f) harmonics behave differently. Therefore, the proper selection of laser spot position also depends on the harmonic order. Note that the scan area in each image may not be the same here.

To gain insight into the influence of the laser spot position on the harmonic signals, we start with calculating the mode shape slopes of the second and third eigenmodes at specified positions along the cantilever. The dynamic oscillation is dominated by the differential equation [37],

[4]
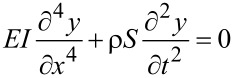


where *E* is the elastic modulus, ρ is the mass density, *S* is the cross-section area and *I* is the area moment of inertia. *x* is the coordinate in the longitudinal direction starting from the fixed end and *y*(*x*) denotes the deflection from the rest position. Solving the above equation, the deflection shape *y_n_*(*x*) of each mode *n* is obtained,

[5]
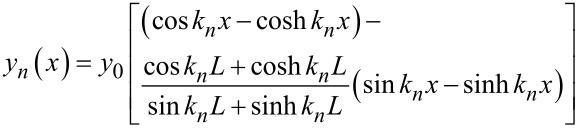


where *y*_0_ is the deflection at the free end, *L* is the cantilever length, and *k_n_* is the wave number, which can be calculated from the characteristic equation cos*k_n_L*cosh*k_n_L* + 1 = 0. Note that the mode shape slope at the laser spot location actually affects the measured cantilever amplitude.

**Figure 6:**
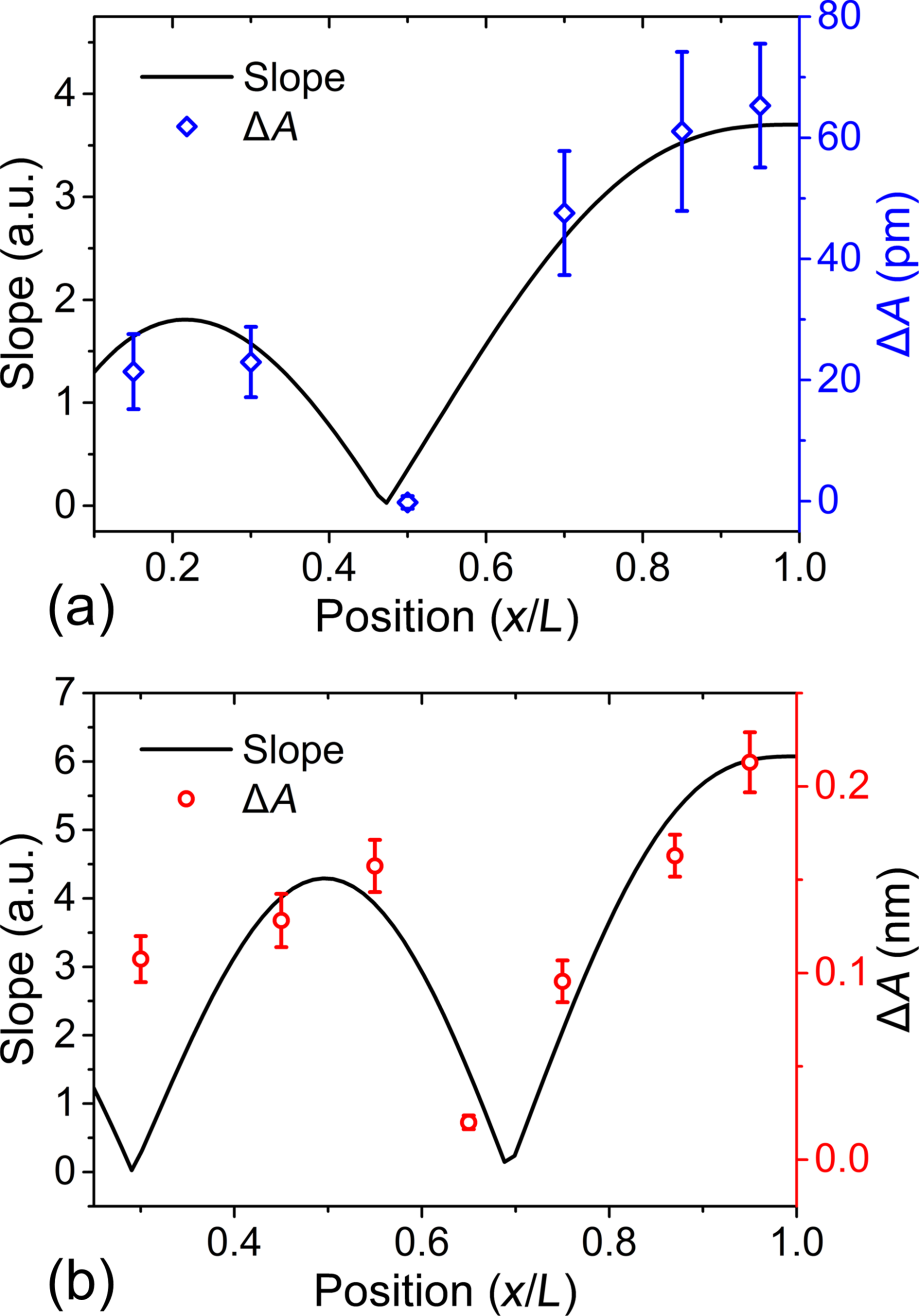
Relation between the harmonic amplitude difference and the slope of the corresponding cantilever mode shape. (a) The 6th harmonic amplitude difference and the mode shape slope of the second eigenmode. (b) The 17th harmonic amplitude difference and the mode shape slope of the third eigenmode.

The evaluated experimental harmonic amplitude difference and the calculated mode shape slope ∂*y*/∂*x* along with the change of the laser spot position (*x*/*L*) by using Equation 5 are plotted in Figure 6. The amplitude at a harmonic frequency near one of the cantilever resonance frequencies roughly scales with the mode shape slope. A laser spot located at the free end can enhance the harmonic signals compared with other locations. Furthermore, positioning a laser spot on the eigenmode slope nodes should be avoided because the harmonic amplitudes will reach to near zero and show almost no sensitivity to material differences.

### Material discrimination and mixture ratio estimation

**Figure 7:**
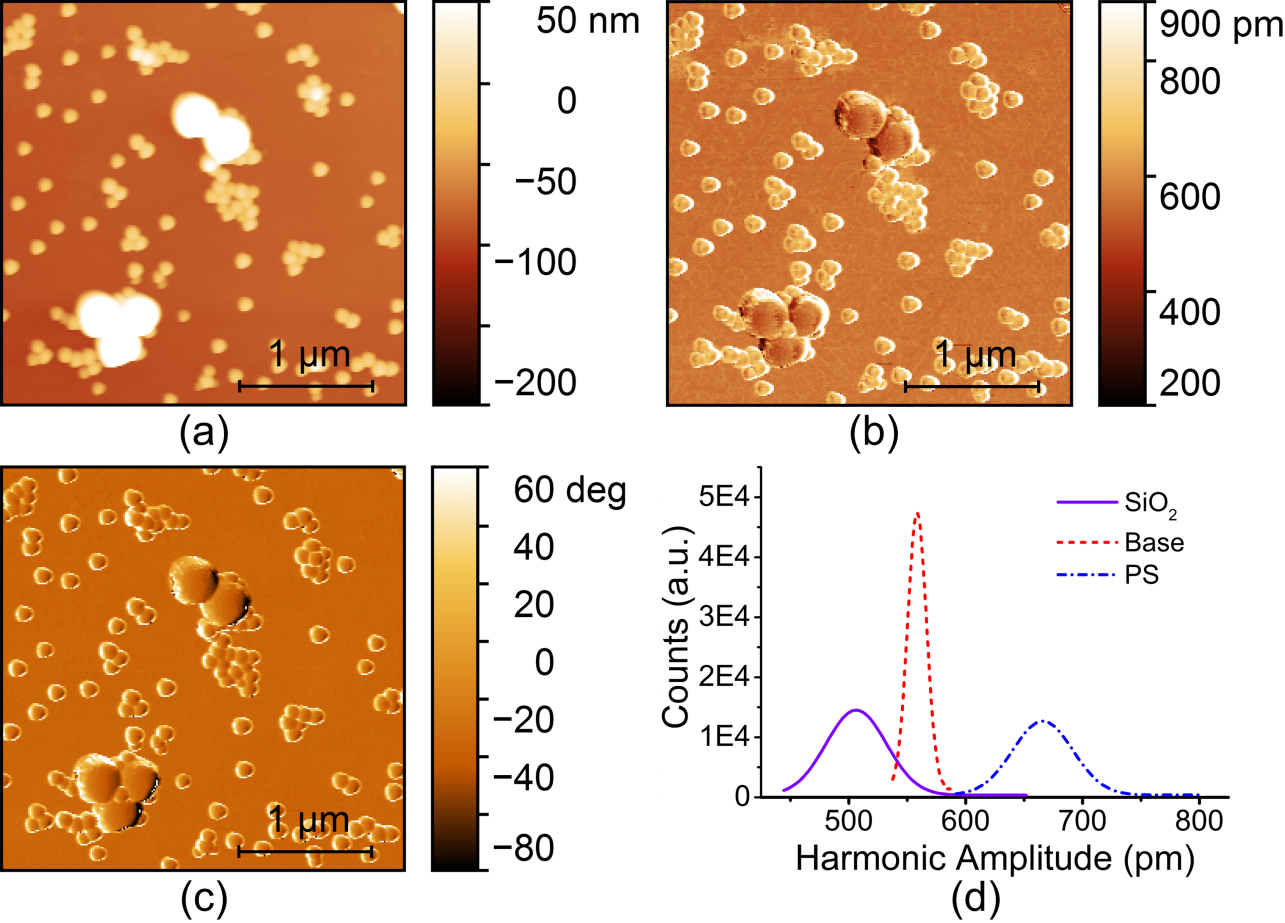
AFM images of mixed PS and SiO_2_ NPs on a silicon substrate. (a) Topography. (b) The 6th harmonic amplitude. (c) Tapping phase. (d) Harmonic amplitude histograms.

After systematic investigations on the influencing factors, we turn to the application of harmonic imaging in material discrimination and mixture ratio estimation of nanocomposite components. The samples were prepared by mixing PS and SiO_2_ NPs and then deposited on a silicon substrate. The harmonic AFM results are presented in Figure 7. For this specimen, the three different materials, PS, SiO_2_ and Si can be distinguished even from the topography because the diameters of the PS and SiO_2_ NPs are obviously different (see Figure 7a). Their nominal diameters are 300 ± 7.5 nm and 100 ± 2.5 nm, respectively. From Figure 7b, the amplitudes for the two materials show a distinguishable difference. The harmonic amplitude on the PS NPs is smaller than that on the SiO_2_ NPs. Three amplitude distribution peaks are found in the histograms demonstrated in Figure 7d. The peaks are centered at 505, 559 and 665 pm, which correspond to SiO_2_ NPs, Si substrate and PS NPs, respectively. The three materials can be clearly separated in the harmonic amplitude images. However, larger harmonic amplitudes do not occur for materials with larger elastic moduli. Such a contrast reversal is due to the deviation in the drive frequency as previously discussed.

The harmonic image was compared with the tapping phase, as shown in Figure 7c. Conventional tapping phase has been verified to have a distinct material discrimination capability. The contrast magnitudes of the 6th harmonic amplitude and tapping phase images calculated by using Equation 1 are 6.31 and 2.94, respectively. The harmonic amplitude contrast is two-fold larger than the tapping phase contrast. The harmonic image contrast can be further enhanced by tailoring the cantilever dynamic spectrum to make the higher-order harmonic frequency and the higher-mode resonance frequency overlap [29]. For instance, the image contrast can be conveniently enhanced up to 6-fold by modifying the cantilever mass distribution [38]. With these optimizations, the amplitude contrast could be one order of magnitude larger than in tapping phase. Harmonic AFM imaging therefore has a substantially stronger capability in discriminating materials. It can be expected that the harmonic signal is able to discriminate between materials with even smaller differences in elastic modulus while the tapping phase signal may fail to distinguish.

**Figure 8:**
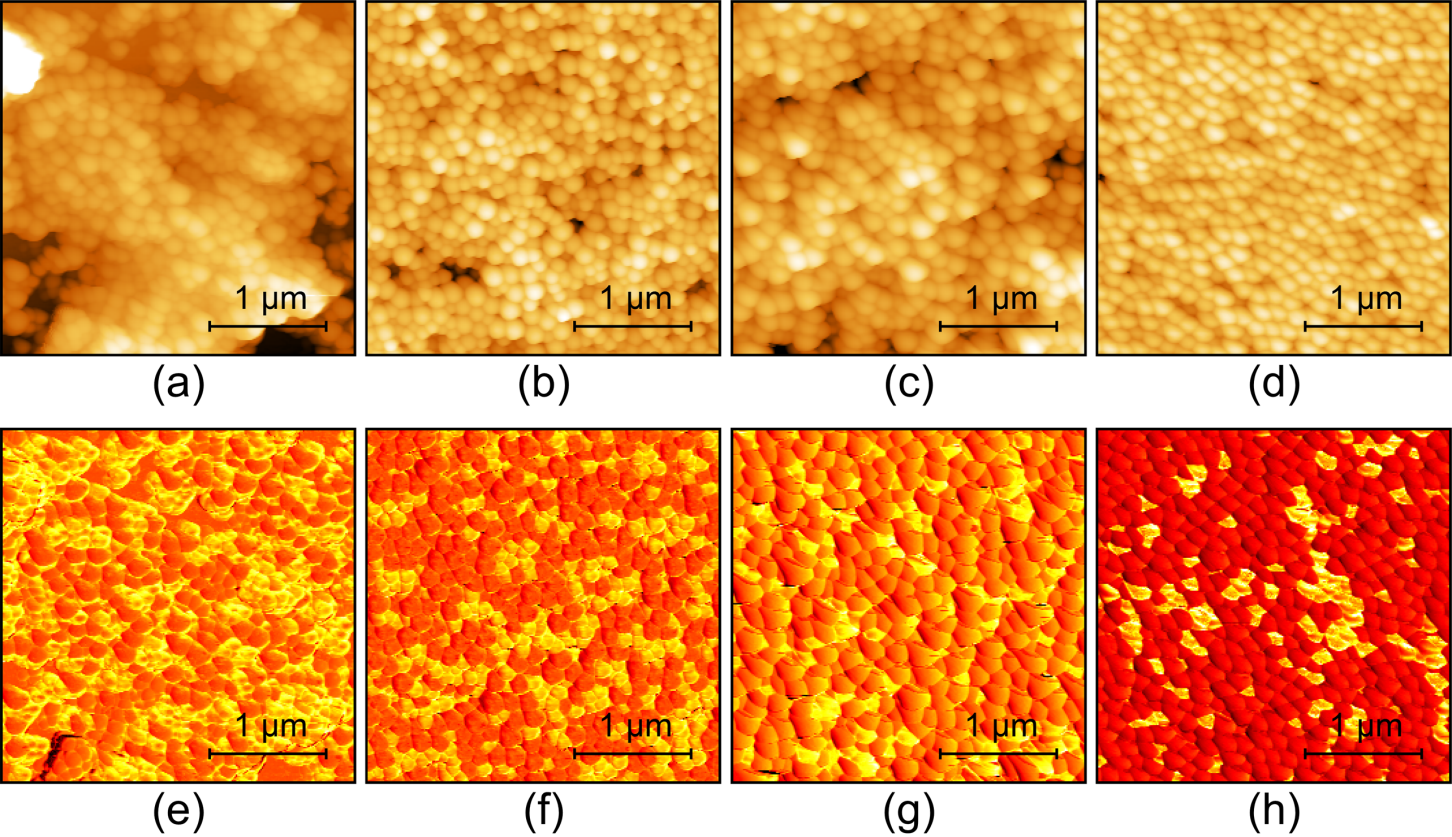
Harmonic AFM imaging of SiO_2_/PS NPs mixed with different ratios. The two types of NPs have the same diameter. (a)–(d) Topography images. (e)–(h) Harmonic amplitude images. From (a)–(d), the nominal mixture ratios are 2:1, 1:1, 1:3 and 1:4, respectively.

To further ascertain the capability of harmonic imaging, we performed another set of experiments on mixing SiO_2_ and PS NPs with the same diameter but different mixture ratios. Figure 8 presents the typical results on four specimens for whom the respective mixture ratios are 2:1, 1:1, 1:3 and 1:4. The topographic images are displayed in Figure 8a–d. The NPs are closely packed and they cannot be unambiguously distinguished because of the same particle shape and size. On the contrary, the 6th harmonic amplitudes presented in Figure 8e–h demonstrate two amplitude magnitudes in the scan area, which correspond to the two kinds of NPs. Careful comparison between the topography and harmonic amplitude images have been conducted. The variation of the surface geometry is found to have little influence on the harmonic amplitude. That is, the amplitude contrast here is mainly attributed to the mechanical properties of the NPs.

**Figure 9:**
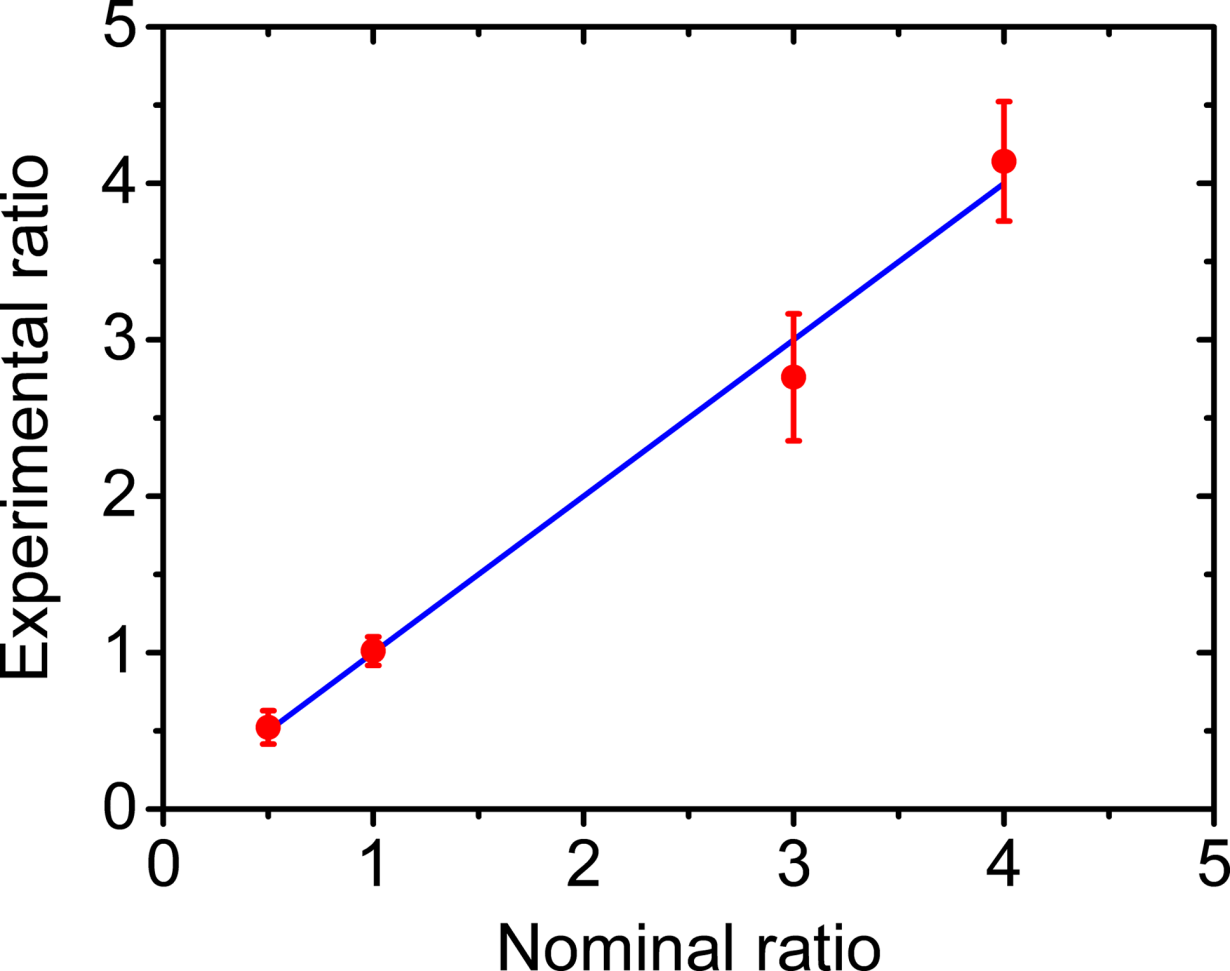
Mixture ratio estimation of the SiO_2_/PS composites.

Since the harmonic images show two obvious amplitude distributions peaks, they can be processed to estimate the mixture ratio of the NPs as demonstrated in Figure 9. To estimate the mixture ratio, we randomly scanned several images with the same scan area at several different positions of each specimen. After acquiring the harmonic amplitude images, the mixture ratios are estimated through classifying and evaluating the image area occupied by the two major amplitude ranges. From the figure, the experimentally measured mixture ratios are close to the nominal ones. From these results, we can conclude that harmonic imaging is an excellent method for visualizing the distribution of different materials and estimating the local mixture ratio.

## Conclusion

In summary, harmonic AFM imaging was employed to discriminate different NPs and estimate their mixture ratio in nanocomposites. Several important factors including the amplitude set-point, drive frequency and laser spot location were investigated systematically. Changing the set-point will induce alternation of the tip–sample interaction forces and thus different harmonic amplitudes. Simulations of the cantilever dynamics were found to correspond well to the experimental observations. Depending on the slight deviation of the drive frequency from the fundamental resonance frequency, harmonic amplitude contrast reversal was found. Such a phenomenon was reasonably interpreted. The laser spot position influences the harmonic amplitude sensitivity, as expected, and proper selection of the laser spot position is crucial to obtain enough signal strength.

Based on these fundamental investigations, harmonic AFM was adopted to discriminate different material components and estimate the mixture ratio of nanocomposite samples that were composed of several kinds of NPs of almost the same diameter. It was shown that AFM higher harmonic imaging offers improved information on the NP distribution than other methods including topography and tapping phase. The results show that higher harmonic imaging is an excellent tool for distinguishing between different materials with satisfactory spatial resolution and mechanical sensitivity. Furthermore, the distribution of material components and their mixture ratio can be determined accurately. Such characteristics validate harmonic AFM as a powerful method for applications in numerous research fields such as material science and nanobiology.

## Experimental

The harmonic imaging experiments were performed on a commercial AFM (MFP-3D Origin, Asylum Research, Santa Barbara, CA). The operation was similar to conventional tapping mode. An extra lock-in amplifier was adopted to analyze the amplitude and phase signals at an integer multiple of the drive frequency. Usually, the drive frequency was selected at or close to the fundamental resonance frequency. However, a certain deviation from the free resonance frequency may exist owing to the measurement error or environmental fluctuation during the resonance spectrum sweeping. For the experiments, ContAl-G cantilevers (BudgetSensors, Innovative Solutions Bulgaria Ltd., Bulgaria) were used. Prior to the measurements, the spring constant of the cantilevers was determined (typically 0.24 N/m) by using the thermal calibration method [39]. For amplitude calibration the inverse optical lever sensitivity in units of nm/V was determined via a noncontact method proposed by M. J. Higgins et al. [36]. The noise level of the AFM optical detector was less than 60 μV, which corresponds to an amplitude noise of approximately 10 pm. The lowest harmonic amplitude measured was quite close to this level, but it could still be unambiguously determined. Other harmonic amplitude magnitudes were generally larger than the noise.

Three kinds of samples were employed for the harmonic AFM measurements. The first is a polymer blend composed of PS and LDPE. This sample is used to study the influencing factors in harmonic imaging. The second is a mixture of PS and SiO_2_ NPs. The nominal diameters of PS and SiO_2_ are 300 ± 7.5 nm and 100 ± 2.5 nm, respectively. Two solutions containing the same concentration of these two kinds of NPs were mixed and diluted with deionized water. To avoid agglomeration, ultrasonic dispersion was applied for at least 5 min. After that, a drop of the solution was deposited on a silicon substrate and dried under natural air conditions in a clean room. For this sample, the two types of particles should also be easily distinguishable from the topography, which is suitable for the verification of the capability of harmonic AFM in discriminating materials. The third kind of sample is a mixture of monodisperse PS NPs and SiO_2_ with the same diameter of about 100 nm. The mixture ratios (SiO_2_/PS) were controlled to be 2:1, 1:1, 1:3 and 1:4, respectively. For these samples, the topographic images show no difference between the two types of NPs. They are prepared to examine harmonic imaging for estimating the mixture ratios quantitatively, which are not available in analyzing other AFM signal channels.

## References

[1] Paul D R, Robeson L M (2008). Polymer.

[2] Balazs A C, Emrick T, Russell T P (2006). Science.

[3] Chatterjee S, Nafezarefi F, Tai N H, Schlagenhauf L, Nüesch F A, Chu B T T (2012). Carbon.

[4] Kaegi R, Ulrich A, Sinnet B, Vonbank R, Wichser A, Zuleeg S, Simmler H, Brunner S, Vonmont H, Burkhardt M (2008). Environ Pollut.

[5] Sahin O, Magonov S, Su C, Quate C F, Solgaard O (2007). Nat Nanotechnol.

[6] Müller D J, Dufrêne Y F (2008). Nat Nanotechnol.

[7] Raman A, Trigueros S, Cartagena A, Stevenson A P Z, Susilo M, Nauman E, Contera S A (2011). Nat Nanotechnol.

[8] Garcia R, Proksch R (2013). Eur Polym J.

[9] Herruzo E T, Perrino A P, Garcia R (2014). Nat Commun.

[10] Noh H, Diaz A J, Solares S D (2017). Beilstein J Nanotechnol.

[11] Magonov S N, Elings V, Whangbo M-H (1997). Surf Sci.

[12] Kuznetsova T G, Starodubtseva M N, Yegorenkov N I, Chizhik S A, Zhdanov R I (2007). Micron.

[13] Yuya P A, Hurley D C, Turner J A (2008). J Appl Phys.

[14] Bar G, Thomann Y, Brandsch R, Cantow H-J, Whangbo M-H (1997). Langmuir.

[15] Garcıa R, Pérez R (2002). Surf Sci Rep.

[16] Rabe U, Amelio S, Kester E, Scherer V, Hirsekorn S, Arnold W (2000). Ultrasonics.

[17] Stan G, Cook R F (2008). Nanotechnology.

[18] Hillenbrand R, Stark M, Guckenberger R (2000). Appl Phys Lett.

[19] Stark R W, Heckl W M (2003). Rev Sci Instrum.

[20] Balantekin M, Atalar A (2005). Phys Rev B.

[21] Balantekin M, Atalar A (2005). Appl Phys Lett.

[22] Loganathan M, Bristow D A (2014). Rev Sci Instrum.

[23] Preiner J, Tang J, Pastushenko V, Hinterdorfer P (2007). Phys Rev Lett.

[24] Krisenko M O, Cartagena A, Raman A, Geahlen R L (2015). Biochemistry.

[25] Schuh A, Bozchalooi I S, Rangelow I W, Youcef-Toumi K (2015). Nanotechnology.

[26] Gramazio F, Lorenzoni M, Pérez-Murano F, Trinidad E R, Staufer U, Fraxedas J (2017). Beilstein J Nanotechnol.

[27] Sahin O, Yaralioglu G, Grow R, Zappe S F, Atalar A, Quate C, Solgaard O (2004). Sens Actuators, A.

[28] Hu S, Raman A (2007). Appl Phys Lett.

[29] Zhang W, Chen Y, Chu J (2017). Sens Actuators, A.

[30] Forchheimer D, Forchheimer R, Haviland D B (2015). Nat Commun.

[31] Basak S, Raman A (2007). Appl Phys Lett.

[32] Fraxedas J, Pérez-Murano F, Gramazio F, Lorenzoni M, Rull Trinidad E, Staufer U (2015). Proc SPIE.

[33] Tamayo J, García R (1996). Langmuir.

[34] Giessibl F J (1997). Phys Rev B.

[35] Ma C, Chen Y, Wang T (2015). AIP Adv.

[36] Higgins M J, Proksch R, Sader J E, Polcik M, Endoo S M, Cleveland J P, Jarvis S P (2006). Rev Sci Instrum.

[37] Rabe U, Janser K, Arnold W (1996). Rev Sci Instrum.

[38] Zhang W, Chen Y, Chu J (2017). Rev Sci Instrum.

[39] Hutter J L, Bechhoefer J (1993). Rev Sci Instrum.

